# How the coronavirus disease 2019 pandemic changed the patterns of healthcare utilization by geriatric patients and the crowding: a call to action for effective solutions to the access block

**DOI:** 10.1007/s11739-021-02732-w

**Published:** 2021-06-09

**Authors:** Gabriele Savioli, Iride Francesca Ceresa, Viola Novelli, Giovanni Ricevuti, Maria Antonietta Bressan, Enrico Oddone

**Affiliations:** 1 Emergency Department, Fondazione IRCCS Policlinico San Matteo, Pavia, Italy; 2 PhD School in Experimental Medicine, Department of Clinical-Surgical, Diagnostic and Pediatric Sciences, University of Pavia, Pavia, Italy; 3 Medical Direction, Fondazione IRCCS Policlinico San Matteo, Pavia, Italy; 4 Department of Drug Science, University of Pavia, Italy and, Saint Camillus International, University of Health Sciences, Rome , Italy; 5 Department of Public Health, Experimental and Forensic Medicine, University of Pavia, Pavia, Italy

**Keywords:** Crowding, Coronavirus disease, Pandemic, Emergency department, Access block, Exit block, Emergency care utilization

## Abstract

The geriatric population constitutes a large slice of the population of Western countries and a class of fragile patients, with greater deaths due to COVID-19. The patterns of healthcare utilization change during pandemic disease outbreaks. Identifying the patterns of changes of this particular fragile subpopulation is important for future preparedness and response. Overcrowding in the emergency department (ED) can occur because of the volume of patients waiting to be seen, delays in patient assessment or treatment in the ED, or impediments to leaving the ED once the treatment has been completed. *O*vercrowding has become a serious and growing issue globally, which represents a serious impediment to healthcare utilization. To estimate the rate of ED visits attributable to the outbreak and guide the planning of strategies for managing ED access or after the outbreak of transmittable respiratory diseases. This observational study was based on a retrospective review of the epidemiological and clinical records of patients aged > 75 years who visited the Foundation IRCCS Policlinic San Matteo during the first wave of COVID-19 outbreak (February 21 to May 1, 2020; pandemic group). The analysis methods included estimation of the changes in the epidemiological and clinical data from the annual baseline data after the start of the COVID-19 pandemic. Outcome measures and analysis: Primary objective is the evaluation of ED admission rate change and ED overcrowding. Secondary objectives are the evaluation of modes of ED access by reason and triage code, access types, clinical outcomes (such as admission and mortality rates). During the pandemic, ED crowding increased dramatically, although the overall number of patients decreased, in the face of a percentage increase in those with high-acuity conditions, because of changes in patient management that have prolonged length of stay (LOS) and increased rates of access block. Overcrowding during the COVID-19 pandemic can be attributed to the Access Block. Access Block solutions are hence required to prevent a recurrence of crowding to any new viral wave or new epidemic in the future.

**Supplementary Information:**

The online version contains supplementary material available at 10.1007/s11739-021-02732-w.

## Introduction

Coronavirus disease 2019 (COVID-19) is an acute respiratory infectious disease that is caused by the novel coronavirus severe acute respiratory syndrome coronavirus 2 (SARS-CoV-2). SARS-CoV-2 is dissimilar from other coronaviruses that usually spread among humans and cause the common cold. The first confirmed case of pneumonia caused by this novel coronavirus was reported at the end of 2019 (WHO, World Health Organization 2019). COVID-19 is particularly pathogenic in humans and associated with high mortality rates. A large percent of patients develop severe disease and experience poor outcomes, especially the elderly [1]. The first wave of COVID-19 outbreak in Italy was reported in Codogno, a municipality of 15,978 inhabitants of the province of Lodi, on February 21, 2020, in Lombardia, near Milan. After a few cases were registered in our hospital, the outpatient clinic was closed, the “regular” patients were discharged or transferred to other wards, and a section of the hospital was transformed into a sub-intensive care ward [2]. To divide the pathways as well as to isolate patients with known or suspected COVID-19 infection, a separate section of the emergency room was created in the infectious disease building of the hospital.

Chronic obstructive pulmonary disease (COPD) is usually prevalent among the elderly population, which has a high impact on the quality of life, morbidity, and mortality [3]. Viruses that cause respiratory tract infections (RTIs) can exacerbate chronic lung diseases such as COPD and asthma, which can require visits to the emergency department (ED) and hospitalization [4]. This event places a huge burden on the healthcare services in the primary and secondary settings and it is the reason for most of the variability in the ED visits and hospitalizations associated with cases of RTIs [5]. During an epidemic, viruses can also cause death, as observed for influenza and respiratory syncytial virus [6], mostly among the elderly population. Therefore, identifying viruses and monitoring the severity of their effects are expected to be major scientific and clinical endeavors.

The elderly often suffers from chronic diseases requiring multi-drug therapy, regular checks for their pathologies, and presents with exacerbations requiring access to EDs. The patterns of healthcare utilization by the elderly change during infectious disease outbreaks. Identifying the patterns of changes is important for future preparedness and response. It seems particularly important to observe this change in an elderly subpopulation, because they represent a fragile population, need frequent access to ED for exacerbations of chronic diseases or for new acute events, and are affected more severely by COVID-19. The effects of infectious disease epidemic on healthcare utilization depend on the characteristics of the infection.

Thus, epidemics can have major effects on the healthcare system, including overcrowding.

Overcrowding in the ED can occur because of an increase in the volume of patients waiting to be seen (input), delays in patient assessment or treatment in the ED (throughput), or impediments to leaving the ED once treatment has been completed (output) [7]. ED overcrowding has become a serious and growing concern globally, representing a serious impediment to healthcare utilization. Overcrowding is the product of several internal and external factors, including insufficient access to hospital beds and shortages of the hospital staff. Overcrowding can lead to poor outcomes and prolonged length of stay (LOS). Elderly patients are at a higher risk than younger patients for complications related to hospitalization and long stays in the ED. Several international studies have reported that overcrowding can result in a greater number of adverse events, with increased morbidity and mortality, prolonged LOS, and reduced healthcare quality [8–10].

ED crowding has been extensively discussed for several decades, with various suggestions about interventions to reduce the ED crowding [11–13]. Presently, the most frequent cause of overcrowding is access block. According to the Australasian College for Emergency Medicine, access block is defined as “the situation where patients are unable to gain access to appropriate hospital beds within a reasonable amount of time (≤ 8 h)” and “overcrowding” refers to “the situation where ED function is impeded by the number of patients waiting to be seen, undergoing assessment or treatment, or waiting for departure, which exceeds the physical or staffing capacity of the department” [14].

Although the causative agent and the mode of transmission of COVID-19 have been examined in detail, the effects of the epidemic on the availability of emergency services and ED overcrowding remains under-evaluated. We performed a large retrospective observational study by comparing the demographic and clinical data of patients after the COVID-19 wave with data for patients who visited the ED in the corresponding period in the past 2 years, as well as the period preceding the outbreak. We believe that overcrowding has increased, as measured using throughput and output indices.

The specific hypotheses made were as follows:

regarding the primary objectives:

1. The number of attenders among the elderly at the ED decreased regardless of the age groups (75–80; 80–85; 85–90; > 90 years) and sex after the COVID-19 outbreak;
2. Throughput (such as the length of ED stay) and output crowding indices (such as the rate of access block, total access block time, and percent of patients who left without being seen) were changed due to the COVID-19 outbreak;
  Regarding the secondary objectives:
3. The modes of ED access (e.g., ambulance and spontaneous), the codes for priority of medical examination, and the exit codes for severity changes after the outbreak reflected more serious illnesses and patients requiring high-intensity care;
4. Marked reduction in some access types (such as access for minor trauma and minor signs and symptoms) was accompanied by a homogeneous reduction in other access types;
5. Clinical outcomes, such as admission and mortality rates, were changed due to the outbreak (as were the output crowding indices).

The final objectives of this study were to estimate the rate of ED visits attributable to the outbreak and guide the planning of strategies for managing ED access or after the outbreak of transmittable respiratory diseases.

## Methods

### Study design

This observational study was based on a retrospective review of the epidemiological and clinical records of patients aged > 75 years visiting the Foundation IRCCS Policlinic San Matteo during the first wave of COVID-19 outbreak (February 21, 2020 to May 1, 2020; pandemic group). Data were extracted using the PiEsse software. The methods of evaluation included the estimation of the changes in the epidemiological and clinical data from the annual baseline data after the start of the COVID-19 pandemic.

Data were provided directly by the San Matteo Hospital Foundation, which maintains files on all services provided by its ED. An ad hoc query was performed to obtain the data of interest. The first names and surnames of the patients were replaced with anonymous codes to ensure proper blinding to the patients’ identities. At the time of ED admission, the patients provided their informed consent for the processing of their data for medical and research purposes.

### Endpoints

The primary outcome was aimed at assessing the changes in the use of emergency resources after the COVID-19 outbreak in terms of the ED visits in an elderly subpopulation and the assessment of ED overcrowding through the use of crowding indices such as the length of ED stay, total access block time, and the rate of access block. Meanwhile, the key secondary aim was to define the characteristics of the population that visited our ED during the pandemic, including the gender, age, and method of ED access. Other examined outcomes included the causes of ED visits during the pandemic; the clinical outcomes such as admission and mortality rates.

### Inclusion and exclusion criteria

All elderly patients (aged ≥ 75 years) who visited the ED during the study periods were eligible for inclusion. Ophthalmological and gynecological emergencies, relating to specialist ERs, were excluded.

### Study population

For each patient, the demographic data (gender and age), vital parameters (blood pressure, heart rate, oxygen saturation, Glasgow Coma Scale, and respiratory rate), signs and symptoms, the waiting time, LOS in the ED, mode of ED access, priority codes for medical examination, exit codes for severity, total access block time, and the rate of access block were collected. All medical records were accurately viewed and evaluated, and all computed tomography data were thoroughly reviewed. All collected data were stored on a spreadsheet using the Microsoft Excel program and, subsequently, used for statistical analyses.

The pandemic group in this study consisted of 1911 consecutive patients who accessed the ED between February 20 and May 1, 2020. As the control period, the sum of timespans from January 1, 2018 to May 1, 2018 from January 1, 2019 to May 1, 2019 and from January 1, 2020 to February 20, 2020 (12,537 people) were used.

### Measurement of crowding

Several indices have been proposed to measure crowding [15, 16]; the most common ones can be grouped as follows:

- Input crowding indices: waiting times, the number of patients visiting the ED, and disease severity and complexity (e.g., the number of patients at each acuity level), the number of people who left without being seen (LWBS).
- Throughput crowding indices: LOS
- Output crowding indices: the mean number or percent of admissions, patients in the ED (number or percent), access block and boarding (the mean number or percent of patients who experienced it), and access block or boarding times (such as the total access block time).

These indices have already been widely validated in the past studies [17, 18].

The “waiting time” can be defined as the total time from the time of initial registration/triage to first being seen by a doctor. The “process time” is the time from the medical contact in ED to the medical decision (for admission, transfer or discharge). The overall LOS in the ED is the time from the arrival at triage or registration until discharge or transfer to another ward. This variable reflects the total patient experience, including care and waiting. Access block can be defined as a > 8-h duration in the ED from the time of presentation to admission [19]. The total access block time thus represents the duration of access block, is, therefore, the total residence time of all patients over the initial 8 h (total LOS-8 of each patient) [20]. Boarding can be defined as a > 6-h duration in the ED from the time of medical examination to admission [21, 22]. Thus, the boarding time represents the duration of boarding, that is the time from end of medical assessment to hospital admission [17–22].

### Statistical analysis

Statistical analyses were conducted using appropriate logistic multivariate regression models to test the association between time variables, while accounting for crowding and the pandemic period. Continuous variables were described as mean and interquartile range, while qualitative variables were expressed as the number of observations and appropriate proportions. Comparisons between the two groups of continuous variables were made with the non-parametric Mann–Whitney test, according to their non-normal distributions, while associations between the qualitative variables were studied with χ^2^ test. Moreover, the test of proportions was used to examine the differences in ED mortality between the two periods. The significance level was set at alpha 0.05 (statistical significance at *p* < 0.05), and all tests were two tailed.

The analyzes were conducted with the STATA software (version 14; Stata Corporation, College Station, TX, USA, 2015). The study was submitted to the ethics committee (*n* 20200114609).

## Results

### Primary endpoints

#### Study population and the use of emergency resources

Our study is focused on 1911 consecutive patients, aged 75 or older, who accessed the ED between February 21, 2020 and May 1, 2020. This group of patients was compared to 12,537 patients, aged 75 or older, who accessed the ED during non-pandemic periods in 2018, 2019 and 2020.

During the pandemic, we observed a substantial decline of approximately 25% in the volume of patients visiting the ED when compared with the corresponding periods of 2018 and 2019 (32/day versus 42/day; *p* < 0.001), considering aggregate data for both genders. We also recorded a reduction of approximately 25% in the total and daily access when compared with the corresponding periods of 2018 and 2019; the latter witnessed an increase in the number of ED visits related to seasonal influenza (*p* < 0.001).

In our study, the difference in per day visits number observed during the pandemic period was higher for low-intensity care (0.9 vs 0.5, *p* < 0.001, 16.7 vs 11.4 *p* = 0.002, 2.1 vs 1.5 *p* < 0.001, for white, green and yellow white codes, respectively) compared to high-intensity care (14.1 vs 12.3, *p* = 0.287, 1.0 vs 1.3, *p* < 0.001, for yellow and red codes, respectively).

### Characteristics of patients who visited our ED during the pandemic

**Fig. 1:**
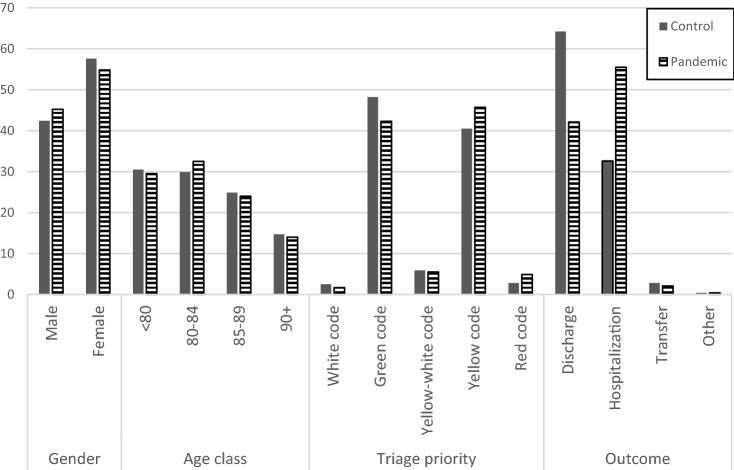
Principal features of control and pandemic groups. Data in percentages. The considered pandemic period spreads from February 21, 2020 to May 1, 2020, while as control period was used the sum of timespan from January 1, 2018 to May 1, 2018, from January 1, 2019 to May 1, 2019 and from January 1, 2020 to February 20, 2020.

Regardless of the gender, the number of ED visits was lower during the pandemic period than during the other periods (26.9 vs. 34.7 visits per day). During the pandemic, a reduction in female prevalence has been observed (57.6% vs. 54.8%, for non-pandemic and pandemic period, respectively, Table 1, Fig. 1).

**Table 1:** Principal personal and Emergency Department presentation features of patients included in the study, by period of observation

Period^a^			
	Control (%)	Pandemic (%)	*p*^b^
Gender			
Male	5,319 (42.4)	864 (45.2)	
Female	7,218 (57.6)	1.047 (54.8)	0.02
Age class			
< 80	3,827 (30.5)	564 (29.5)	
80–84	3,749 (29.9)	622 (32.5)	
85–89	3,115 (24.9)	458 (24.0)	
90 +	1,846 (14.7)	267 (14.0)	0.14
Transport			
Personal	4,907 (39.1)	397 (20.8)	
Ambulance	3,462 (27.6)	711 (37.2)	
MSB	3,817 (30.5)	748 (39.1)	
MSA	312 (2.5)	53 (2.7)	
Other	39 (0.3)	2 (0.1)	< 0.001
Triage priority			
White code	318 (2.5)	32 (1.7)	
Green code	6,033 (48.2)	807 (42.3)	
Yellow-white code	740 (5.9)	105 (5.5)	
Yellow code	5,077 (40.5)	873 (45.7)	
Red code	255 (2.8)	93 (4.9)	< 0.001
Outcome			
Discharge	8,051 (64.2)	804 (42.1)	
Hospitalization	4,085 (32.6)	1,060 (55.5)	
Transfer	348 (2.8)	40 (2.1)	
Other	53 (0.4)	7 (0.4)	< 0.001

*MSA* ambulance with doctor, *MSB* ambulance with nurse

^a^The considered pandemic period spreads from February 21, 2020 to May 1, 2020, while as control period was used the sum of timespan from January 1, 2018 to May 1, 2018, from January 1, 2019 to May 1, 2019 and from January 1, 2020 to February 20, 2020

^b^χ^2^ test

We divided the population into different age groups, as follows: 75–80, 80–85, 85, 90, and ≥ 90 years. During the pandemic, we observed reductions in the number of ED visits among all age groups, without any statistically significant differences (*p* < 0.14; Table 1, Fig. 1). Also, when the number of visits per day was taken into consideration the reduction of ED accesses was confirmed (Table S2).

Meanwhile, the mode of arrival to the ED markedly changed during the pandemic period. On the other hand, 39.1% of the patients typically arrived to the ED using their own transportation prior to the pandemic, and only 20.8% of the patients arrived using autonomous means of transportation during the pandemic (*p* < 0.001; Table 1).

The pandemic period witnessed an overall decrease in the number of patients, but a percent greater need for medical care and a higher intensity of care. Conversely, fewer patients required low-intensity care (*p* < 0.001; Table 1, Fig. 1).

During the pandemic, the vital signs of the patients at admission only modestly deteriorated (Table 2). In fact, the main alteration occurred in a more marked percent of patients with desaturation (< 95%).

**Table 2:** Principal heart function parameters at presentation for patients include in the study, by period of observation

Period^a^			
	Control	Pandemic	*p*
Heart rate			
Observations	10,246	1,703	
Mean (bpm)	81.2	82.7	
IQR	70–90	70–92	< 0.001^b^
Heart rate > 110 bpm			
No	9,517 (75.9%)	1,557 (81.5%)	
Yes	3,020 (24.1%)	354 (18.5%)	< 0.001^b^
O_2_ saturation			
Observations	10,194	1,697	
Mean (%)	95.9	94.9	
IQR	95–98	94–98	< 0.001^b^
O_2_ saturation < 95%			
No	10,125 (80.8%)	1,359 (71.1%)	
Yes	3,412 (19.2%)	552 (28.9%)	< 0.001^b^
Systolic blood pressure			
Observations	10,328	1,714	
Mean (mmHg)	143.0	140.7	
IQR	125–160	120–160	< 0.001^b^
Systolic blood pressure < 90 mmHg			
No	12,377 (98.4%)	1,871 (97.9%)	
Yes	200 (1.6%)	40 (2.1%)	0.113^b^

^a^The considered pandemic period spreads from February 21, 2020 to May 1, 2020, while as control period was used the sum of timespan from January 1, 2018 to May 1, 2018, from January 1, 2019 to May 1, 2019 and from January 1, 2020 to February 20, 2020

^b^Mann–Whitney testm, χ^2^ test

^c^Interquartile range

#### Crowding indices

##### Input indices

**Fig. 2:**
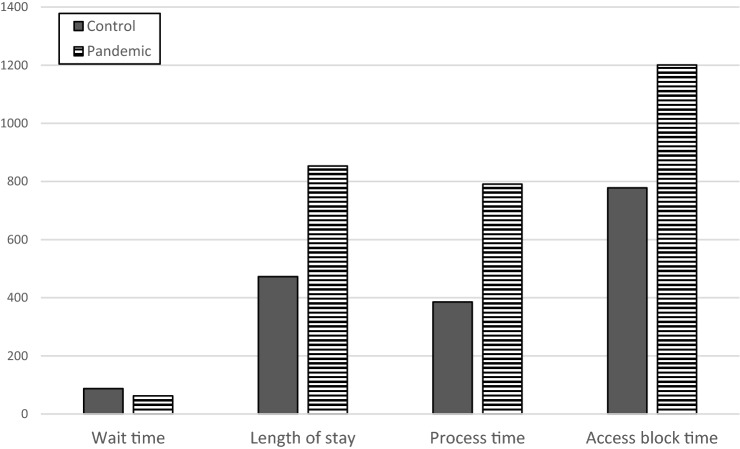
Effect of pandemic on principal times in ED treatment. Data in minutes The considered pandemic period spreads from February 21, 2020 to May 1, 2020, while as control period was used the sum of timespan from January 1, 2018 to May 1, 2018, from January 1, 2019 to May 1, 2019 and from January 1, 2020 to February 20, 2020.

During the pandemic period, a global reduction in the waiting time (from arrival in ED to visit) has been recorded (*p* < 0.001;Tables 3, Fig. 2). Analyzing the various priority codes revealed that the reduction was statistically significant for the visit for green and yellow triage codes, while this reduction was not statistically significant for yellow–white code and only a small insignificant increase in waiting time was observed for white and red codes (Table S1).

**Table 3:** Selected time variables accounting for crowding, by period

	Period^a^	Observations	Mean	Interquartile range	*p*^b^
Wait time (min)					
Control		12,536	87.4	22.2–129.5	
Pandemic		1,911	62.4	12.6–87.4	< 0.001
LOS^c^ (min)					
Control		12,536	472.5	166.6–509.5	
Pandemic		1,911	853.1	220.2–1099.6	< 0.001
Process time (min)					
Control		12,536	385.2	96.3–405.3	
Pandemic		1,911	790.7	154.3–1012.8	< 0.001
Access block total time^c^ (min)					
Control		1,775	778.2	252.5–1053.2	
Pandemic		616	1200.6	341–1434.9	< 0.001

^a^The considered pandemic period spreads from February 21, 2020 to May 1, 2020, while as control period was used the sum of timespan from January 1, 2018 to May 1, 2018, from January 1, 2019 to May 1, 2019 and from January 1, 2020 to February 20, 2020

^b^Mann-Whitney test, calculated only on hospitalized patients

^c^*LOS*: Length of stay in emergency department

##### Throughput indices

During the pandemic, the time spent in the ED increased, both for the process time and for LOS in ED (*p* < 0.001;Table 4, Fig. 2). The prolongation of LOS in the pandemic period compared with that in the control period showed statistically significant difference after adjustment for age, gender, priority code, and the need for moderate-to-high-intensity care (*p* < 0.001; Table 4).

**Table 4:** Risk of overtime for selected time variables accounting for crowding, by period

Period^a^	OR^b^	95% confidence interval	*p*
LOS			
Control	1.00 (Ref.)	–	
Pandemic	2.28	2.00–2.60	< 0.001
Boarding			
Control	1.00 (Ref.)	–	
Pandemic	2.72	2.43–3.06	< 0.001
Access block			
Control	1.00 (Ref.)	–	
Pandemic	2.47	2.21–2.76	< 0.001

*LOS* Length Of Stay

^a^The considered pandemic period spreads from February 21, 2020 to May 1, 2020, while as control period was used the sum of timespan from January 1, 2018 to May 1, 2018, from January 1, 2019 to May 1, 2019 and from January 1, 2020 to February 20, 2020

^b^Odds ratios (OR) estimated by multiple regression analysis adjusted by age, gender, priority code at triage, presence of fever or respiratory symptoms and need for moderate-to-high–intensity care

##### Output indices

During the pandemic, all crowding output indices increased, especially with respect to the rates of boarding and access block and the total boarding and access block times (*p* < 0.001; Table s  4and 5, Fig. 2). The increased frequencies of boarding (percent and total time) and access block (percent and total time) in the pandemic period when compared with that during the control periods remained statistically significant even after adjustment for age, gender, priority code, and the need for moderate-to-high-intensity care (*p* < 0.001; Table 3, 4).

### Secondary outcomes

#### Various causes (in percent) of ED visits

During the pandemic, fewer patients visited the ED, especially for minor medical issues (e.g., dermatological conditions and otolaryngological diseases) and minor trauma. The incidence of major trauma decreased slightly without reaching statistical significance. Conversely, access for fever and respiratory symptoms decreased only a little in number, but increased in percent. The same trend was noted for visits related to neurological symptoms (Table 5).

**Table 5:** Selected access to Emergency Department causes for patients included in the study, by period of observation

	Period^a^		
	Control	Pandemic	*p*^b^
Minor medical issues			
No	10,767 (85.9%)	1,709 (89.4%)	
Yes	1,770 (4.1%)	202 (10.6%)	< 0.001^b^
Minor trauma			
No	11,359 (90.6%)	1,791 (93.7%)	
Yes	1,178 (9.4%)	120 (6.3%)	< 0.001^b^
Major trauma			
No	12,514 (99.8%)	1,910 (99.9%)	
Yes	23 (0.2%)	1 (0.1%)	0.358^c^
Disease with fever			
No	12,100 (96.5%)	1,640 (85.8%)	
Yes	473 (3.5%)	271 (14.2%)	< 0.001^b^
Respiratory symptoms			
No	10,668 (85.1%)	1,550 (81.1%)	
Yes	1,869 (14.9%)	361 (18.9%)	< 0.001^b^
Thoracic pain			
No	11,520 (91.9%)	1,769 (92.6%)	
Yes	1,017 (8.1%)	142 (7.4%)	0.307^b^
Neurologic disease			
No	11,249 (89.7%)	1,673 (87.6%)	
Yes	1,288 (10.3%)	238 (12.4%)	0.004^b^

^a^The considered pandemic period spreads from February 21, 2020 to May 1, 2020, while as control period was used the sum of timespan from January 1, 2018 to May 1, 2018, from January 1, 2019 to May 1, 2019 and from January 1, 2020 to February 20, 2020

^b^χ^2^ test,

^c^Fisher test

#### Clinical outcomes

During the pandemic, geriatric patients showed worse percent exit codes and hospitalization rates (*p* < 0.001; Table 1). The percent need for hospitalization increased from approximately 32.6–55.5% (*p* < 0.001; Table 1, Fig. 1). Importantly, although the total number of ED visits decreased, the number of deaths increased. In fact, we recorded 115 deaths between February 21, 2020 and May 1, 2020 (pandemic), while the mean number of deaths in the corresponding periods of 2018 and 2018 was 37.5. Considering the difference in the patient numbers (6729 in pandemic period, and a mean value of 12,403 in 2018 and 2019), we estimated a mortality rate in ED of 1.71/100 patients during the pandemic and of 0.31/100 patients (*p* < 0.001) for previous corresponding periods (36 and 39 deaths for 2018 and 2019, respectively).

## Discussion

### Assessment of emergency resource use during the COVID-19 epidemic

This study evaluated the changes in the utilization of emergency care throughout the first wave of COVID-19 pandemic by geriatric patients in a single ED based in Lombardy, a region in the northeastern Italy and one of the most severely affected COVID-19 areas. There have been radical changes in the manner in which emergency care is accessed in this area. We recorded a sharp reduction in the number of autonomously transported patients and an increase in those transported to the ED by the territorial emergency service.

The patterns of medical service used during infectious disease outbreaks can vary based on the characteristics of the infection, including the infectivity and lethality of the disease. In the 2003 SARS epidemic in Taiwan, the medical service utilization declined owing to the perceived risk of nosocomial transmissions [23]. However, in the 2009 influenza H1N1 pandemic, which was typified by high infectivity but low-case fatality rates, the emergency care utilization had increased [24]. In 2015, South Korea (hereafter referred to as “Korea”) experienced an epidemic of the Middle East respiratory syndrome (MERS). The Korean citizens avoided healthcare facilities because of the fears of potential nosocomial transmission of this unfamiliar contagious disease. Development and spread of such fears affected the entire society, and, as a result, all healthcare utilization rapidly decreased [25, 26].

Thus, epidemics can have different effects on patient behavior depending on the mortality rates and the emotional impact of the epidemic itself on the population. On one hand, epidemics with high-mortality rates are likely to reduce the demands on the health system, but increase the number of patients with serious diseases. On the other hand, epidemics with lower mortality rates and lower emotional effects can result in greater healthcare utilization [23, 27].

Both SARS and H1N1 influenza caused global panics, but the patterns of healthcare utilization during these outbreaks were totally opposite. Healthcare utilization decreased during the 2003 SARS epidemic because of concerns over nosocomial infection, whereas it increased explosively during the 2009 H1N1 influenza pandemic because of the excessive fear of influenza itself [23]. During the MERS epidemic, healthcare utilization decreased, because the main transmission route was nosocomial infection and the resultant mortality rate was high [23, 28].

Geriatric patients are particularly vulnerable to this virus and, often, already suffer from pulmonary and cardiovascular comorbidities. However, only a few studies have investigated the use of emergency services by the elderly population in various pandemics.

There have been numerous appeals by the civil authorities to the elderly population to reduce their social activities as much as possible, avoiding all those events that are not essential. The high number of mortalities associated with the COVID-19 pandemic has spurred civil authorities to implement measures to contain the virus. Accordingly, “Red zones” have been created, which are areas with restrictions on citizens’ movements, business closures, and working from home, when possible. It is believed that newscasts that constantly updated the spread and mortality of COVID-19 likely promoted apprehension among the population.

As observed in past studies that examined changes in healthcare utilization according to disease severity, the reduction in emergency care utilization was most prominent for low-acuity conditions (i.e., non-urgent, minor emergency, and emergency requiring low-intensity care) [23, 27, 31, 32]. Thus, emergency services requiring high-intensity care remained substantially unchanged during the pandemic, with a slight increase of red codes.

More precisely, there was only a slight reduction in the absolute number of patients with red code and yellow code, with an increase in their percentage representativeness. That there has been an increase in more serious codes is in line with what emerged from a maxi-emergency simulation conducted on Italian territory by our study group. The same also highlighted how the geriatric population presented greater management criticality in cases of maxi-emergency [29]. It is our opinion that in cases of maxi-emergency, not only of an infectious type such as an epidemic or a pandemic, but also of a physical, chemical or massive influx of injured, we should expect an increase in the high priority codes for medical examination and a greater need for medium-to-high-intensity care beds.

Last but not least a selection of ED visits—independent of patients' choice or fears—was probably performed for several milder cases by GPs and Prehospital Emergency Care.

### Various causes of ED visits

During the ongoing pandemic, there has been a net reduction in some reasons for ED visits, such as for minor trauma or minor medical issues, which confirms the reduction in the number of low-acuity visits. The percent of patients with febrile symptoms at home was much higher during the pandemic period.

Patients decided to employ medical care after considering the risks and benefits. During an infectious disease epidemic, patients use medical care when they believe that the benefits of healthcare utilization exceeds the risk of infection [23, 25]. When patients have concerns about nosocomial infections, those with low-acuity diseases are less likely to visit the ED [28]. Visits by patients with low-acuity conditions most strongly decrease when the risk of infection overwhelms the benefits of availing emergency services. A study of the SARS epidemic revealed that the restriction of non-urgent hospital utilization did not increase the resultant mortality and complication rates [30]. These authors accordingly concluded that restricting non-urgent visits is a safe public health strategy for controlling the spread of nosocomial infections and maintaining the hospital surge capacity.

Regarding visits for high-acuity conditions, the mild reduction in access for major trauma can be explained by the fact that our teaching hospital no longer served as the hub for major trauma during the pandemic period. This reduction may also be attributed to the least occurrence of car accidents for the lockdown declared by the government. The fact that the reduction was not statistically significant could be due to the fact that, in any case, the elderly can report major traumas even for minor dynamics and with domestic accidents. The rate of visits for serious conditions did not decline in the same manner. As observed for chest pain and neurological symptoms in this study, the reduction in ED use for high-acuity diseases was expected to be minor during the infectious disease outbreaks. When fears of an epidemic spreads and ED visits decrease, preparations for serious conditions must be focused, so that patients with severe diseases do not face barriers to emergency care. This point also underlines the need to consider “clean” or low-risk infectious pathways for the most serious reasons for ED visits of geriatric patients.

### Crowding indices

#### Causes of crowding

Crowding of EDs has been reported as an issue for several decades now. We found that the input factors play a modest/ambivalent role in crowding in this pandemic situation, whereas throughput and output factors more accurately reflect crowding and the work performed by healthcare providers in this pandemic. The input factors include the number of patients who visit the ED and the severity of diseases, including the risk of infection [7]. The input factors play varying roles, as only some factors affect crowding during this pandemic. We observed a reduction in the crude volume of emergency visits. However, in this aspect, it must be considered that the elderly constitute a fragile population that require emergency services more than others for the decompensation of chronic diseases. The reduction in all-cause ED access for this subpopulation can also be attributed to the fear of contracting an infection in the hospital. Unless accompanied by an increase in clean territorial visits for checkups and outpatient visits due to the first signs of decompensation of chronic diseases, this reduction could lead to serious consequences on the health of these fragile patients. We also noted a reduction in the waiting time, but a greater number of high-acuity visits. The waiting times were reduced in concert with the number of ED visitors and independently of the crowding.

Our study revealed that the pandemic has placed a tremendous burden globally, which has unprecedentedly raised the need for intensive care beds [1], leading to ED overcrowding. We believe this increment of crowding is attributable to three factors: the discrepancy between the immediate and sudden need for intensive care beds and the number of intensive care beds available on the basis of national and local historical needs; the high number of critically ill geriatric patients who require stabilization before transferring to hospital wards; and the change in the management of all patients caused by the pandemic. It seemed necessary to screen all geriatric patients before their admission to ensure that infected and asymptomatic patients were not admitted to “clean” wards or wards with a low risk of infection. For this purpose, nasal swabs, chest X-ray, and bedside lung ultrasound were obtained from each patient for serological tests, and the patients awaited the results in a specific location separate from other inpatients in the ward. These necessary safety measures prolonged the processing time and LOS, together with requiring frequent sanitation and appropriate use of personal protective equipment by the healthcare professionals. The longer stay of elderly patients in the ED puts them at greater risk of contracting nosocomial infections, mental disorders, and worsening nursing care. Notably, during the study period, the relatives could not enter or assist the patients, for dutiful reasons to contain the pandemic, which created further discomfort among this category of fragile elderly subjects.

Thus, increased rates of boarding and access block were noted to affect all patients during the pandemic, including those who were COVID-19 negative, despite the strong effort to add, during the emergency peak, almost 300 beds for COVID-19 patients, 65 of which were dedicated to providing intensive care.

#### Possible crowding responses

Several past researchers and research communities have developed measures to prevent ED crowding and provide appropriate care for patients receiving emergency care. Interventions were categorized into input, throughput, and output controls [7, 11–14]. In particular, the American College of Physicians (ACEP) recently identified ED boarding and access block as “the primary [causes] of ED crowding” [31]. Previous investigations have addressed the question of whether ED crowding affects the mortality and morbidity rates in the ED. Moreover, several studies and systematic reviews have confirmed the association between ED crowding and increased mortality rates [9–16]. However, the effects of crowding on the quality of care were not examined in this study. However, measures to alleviate crowding and reduce the access block are required to prepare adequate responses for future pandemics.

Until date, emergency preparedness for outbreaks of transmittable respiratory illness has scarcely focused on preventing overcrowding and protecting the staff and patients. Instead, the focus has been on preparing emergency quarantine areas and isolating the admission rooms. Overcrowding provides favorable conditions for transmission among patients in the ED through respiratory droplets, and prior researches have recommended infection control measures, such as case management, isolation, and planning for complex emergencies [9–16, 31].

To improve the practice of boarding patients, the ACEP established a task force to develop a list of low-cost, high-impact solutions [9–16, 31]. One of the key solutions proposed by the ACEP is the use of a full-capacity protocol (FCP). An FCP suggests that, when a patient requires admission to an inpatient unit from the ED and when that unit cannot accommodate the patient because of the lack of available beds, the patient can be admitted to the next most appropriate unit. In an event that appropriate hospital bed utilization has been maximized, select admitted patients boarding in the ED should be transferred to hallways in the inpatient units, instead of in boarding in the ED hallways (i.e., inpatient boarding). Although these patients are not physically present in a room, they can receive care from inpatient physicians and nurse specialists, which would enable ED providers to continue serving new ED patients [28].

This measure has been applied in our hospital by creating ad hoc, high-intensity holding area communicating with the emergency room by utilizing medical and nursing staff from the intensive care unit (ICU) and infectious diseases department. Therefore, nurses and doctors from other departments were also employed (e.g., surgeons, dermatologists, and ophthalmologists). Although this was an effective response, the need for effective solutions for reducing the access block must be reiterated. For instance, in 2003, Asplin et al. [7] advocated to researchers and policy makers to focus their efforts on alleviating this problem. Considering the emergence of pandemics and other emergencies, we must emphasize that “access Block and ED overcrowding have created a dynamic tension and the future of emergency medicine will be determined by the resolution of this conflict” [32]. The authors were convinced that it is necessary to plan territorial assistance paths for the elderly and, if possible, dedicated paths within the EDs for better combating the issue of crowding.

### Clinical outcomes

We recorded that the rates of more serious exit codes and the need for hospitalization were approximately two-fold greater during the study period than otherwise in the control periods. This observation signifies the major impact of the current pandemic on the existing healthcare system [1] as well as emphasizes on the high rates of access block and boarding that has become synonymous with this pandemic. We noted that, a greater need for sudden hospitalization, in this case nearly two-fold greater than the historical requirement, resulted in a more rapid saturation of hospital beds. In addition, patients with greater disease severity require longer hospital stays.

### Limitations

This study had several limitations. First, this analysis was based on the assumption that no meaningful changes in the factors affecting ED utilization other than the COVID-19 pandemic occurred during the study period, which could not be verified. Second, this study only included patients who had visited the ED. The health outcomes of other patients remain uninvestigated. Although the ED visits for the most severe conditions did not decrease, the global reduction in emergency care utilization may have led to complications other than short-term mortality [31]. For example, there has been a reduction in access to acute myocardial infarction and a subsequent increase in delayed diagnosis [33]. Thus, further studies are warranted to investigate the long-term outcomes of reduced emergency care utilization. Third, ED visits associated with major trauma and traffic accidents were not evaluated in this study because our hospital did not serve as the hub for major trauma treatment during the pandemic. Finally, the study was limited by its single-center nature.

## Conclusions

The study identified a reduction in the number of ED visits during the first wave of COVID-19 pandemic, irrespective of the age and gender of the patients, especially for low-acuity conditions. However, geriatric patients who visited the ED more frequently were hemodynamically unstable; moreover, they more commonly exhibited abnormal vital signs and they more frequently required high-intensity care and hospitalization. During the pandemic, ED crowding showed a dramatic increment, primarily because of the increased number of visits by patients with high-acuity conditions, changes in the patient management system with prolonged LOS, and an increase in the rates of boarding and access block.

## Supplementary Information

Below is the link to the electronic supplementary material.

Supplementary file1 (DOCX 15 KB)

Supplementary file2 (DOCX 13 KB)
