# Supplementary material for: How the coronavirus disease 2019 pandemic changed the patterns of healthcare utilization by geriatric patients and the crowding: a call to action for effective solutions to the access block
Source: Intern Emerg Med. 2021 Jun 9;17(2):503–14. doi: 10.1007/s11739-021-02732-w (PMC8188157; doi:10.1007/s11739-021-02732-w)
Supplement: Supplementary file 1 — Supplementary file1 (DOCX 15 KB) [file 11739_2021_2732_MOESM1_ESM.docx]

**Table S1 –** Wait time (min) by priority code at triage and period.

| **Code** | **Period*** | **Observations** | **Mean** | **Standard deviation** | ***p^a^*** |
| --- | --- | --- | --- | --- | --- |
| White code | Control | 318 | 73.7 | 70.77 |  |
|  | Pandemic | 32 | 82.1 | 87.94 | 0.911 |
|  |  |  |  |  |  |
| Green code | Control | 6,032 | 116.1 | 93.33 |  |
|  | Pandemic | 807 | 87.1 | 90.84 | <0.001 |
|  |  |  |  |  |  |
| Yellow-white code | Control | 740 | 44.8 | 53.57 |  |
|  | Pandemic | 105 | 40.1 | 44.53 | <0.001 |
|  |  |  |  |  |  |
| Yellow code | Control | 5,077 | 66.2 | 64.67 |  |
|  | Pandemic | 873 | 47.1 | 53.84 | <0.001 |
|  |  |  |  |  |  |
| Red code | Control | 355 | 7.9 | 8.73 |  |
|  | Pandemic | 93 | 10.5 | 20.91 | <0.001 |

* The considered pandemic period spreads from February 21, 2020 to May 1, 2020, while as control period was used the sum of timespan from January 1, 2018 to May 1, 2018, from January 1, 2019 to May 1, 2019 and from January 1, 2020 to February 20, 2020.

^a^: Mann-Whitney test.
