# Supplementary material for: How the coronavirus disease 2019 pandemic changed the patterns of healthcare utilization by geriatric patients and the crowding: a call to action for effective solutions to the access block
Source: Intern Emerg Med. 2021 Jun 9;17(2):503–14. doi: 10.1007/s11739-021-02732-w (PMC8188157; doi:10.1007/s11739-021-02732-w)
Supplement: Supplementary file 2 — Supplementary file2 (DOCX 13 KB) [file 11739_2021_2732_MOESM2_ESM.docx]

**Table S2**. Comparison between number of visits and number of visits per day, by period.

|  | **Number of visits** |  | **Number of visits per day** |  |
| --- | --- | --- | --- | --- |
|  | Control | Pandemic | Control | Pandemic |
| **Age class** |  |  |  |  |
| <80 | 3827 | 564 | 10.6 | 7.9* |
| 80-84 | 3749 | 622 | 10.4 | 8.8 |
| 85-89 | 3115 | 458 | 8.6 | 6.5* |
| 90+ | 1846 | 267 | 5.1 | 3.8* |

P<0.05, as a result of the proportion test.
